# Myocardial involvement in children with post-COVID multisystem inflammatory syndrome: a cardiovascular magnetic resonance based multicenter international study—the CARDOVID registry

**DOI:** 10.1186/s12968-021-00841-1

**Published:** 2021-12-30

**Authors:** Florence A. Aeschlimann, Nilanjana Misra, Tarique Hussein, Elena Panaioli, Jonathan H. Soslow, Kimberly Crum, Jeremy M. Steele, Steffen Huber, Simona Marcora, Paolo Brambilla, Supriya Jain, Maria Navallas, Valentina Giuli, Beate Rücker, Felix Angst, Mehul D. Patel, Arshid Azarine, Pablo Caro-Domínguez, Annachiara Cavaliere, Giovanni Di Salvo, Francesca Ferroni, Gabriella Agnoletti, Laurent Bonnemains, Duarte Martins, Nathalie Boddaert, James Wong, Kuberan Pushparajah, Francesca Raimondi

**Affiliations:** 1 Department of Pediatric Immunology-Hematology and Rheumatology, Hôpital Necker Enfants Malades, Paris, France; 2 Division of Pediatric Cardiology, Zucker School of Medicine, Cohen Children’s Medical Center of NY, Northwell Health, New York, USA; 3 Pediatric Cardiology, UT Southwestern, Dallas, TX USA; 4 Pediatric Radiology, Hôpital Necker Enfants Malades, Université de Paris, Paris, France; 5 Unité Médico-Chirurgicale de Cardiologie Congénitale et Pédiatrique, Centre de Référence des Maladies Cardiaques Congénitales Complexes-M3C, Hôpital Universitaire Necker-Enfants Malades, Université de Paris, 149, Rue de Sèvres, 75743 Paris, France; 6 Division of Pediatric Cardiology, Thomas P Graham Jr, Vanderbilt University Medical Center, Nashville, TN USA; 7 Department of Pediatrics, Yale University School of Medicine, New Haven, CT USA; 8 Department of Radiology, Yale University School of Medicine, New Haven, CT USA; 9 Cardiovascular Department, ASST Papa Giovanni XXIII, Bergamo, Italy; 10 Radiology Department, ASST Papa Giovanni XXIII, Bergamo, Italy; 11 Division of Pediatric Cardiology, Department of Pediatrics, New York Medical College, Maria Fareri Children’s Hospital at Westchester Medical Center, Valhalla, NY USA; 12 Radiology Department, Hospital Universitario 12 de Octubre, Madrid, Spain; 13 Pediatric Cardiology, Niguarda Hospital, Milan, Italy; 14 Department of Paediatric Cardiology, Evelina London Children’s Hospital, London, UK; 15 Research Department, Rehaklinik Bad Zurzach, Zurzach Care Group, Bad Zurzach, Switzerland; 16 Division of Pediatric Cardiology, University of Texas Health Science Center, Houston, TX USA; 17 Radiology Department, Groupe Hospitalier Paris Saint Joseph, Paris, France; 18 Imagen Pediatrica, Hospital Universitario Virgen del Rocío, Sevilla, Spain; 19 Department of Women’s and Children’s Health, University of Padua, Padua, Italy; 20 Cardiology Department, Regina Margherita Children’s Hospital, Turin, Italy; 21 Paediatric Cardiology, University Hospital of Strasbourg, Strasbourg, France; 22 ICube, Équipe MecaFlu, UMR 7357, University of Strasbourg, Strasbourg, France; 23 Pediatric Cardiology Department, Hospital de Santa Cruz, Centro Hospitalar Lisboa Ocidental, Lisbon, Portugal; 24 Institut Imagine, Paris, France; 25 School of Biomedical Engineering and Imaging Sciences, King’s College London, London, UK; 26 Decision and Bayesian Computation, Computation Biology Department, CNRS, URS 3756, Neuroscience Department, CNRS UMR 3571, Institut Pasteur, Paris, France

**Keywords:** SARS Cov-2 infection, CMR, Acute myocarditis, Children, MIS-C

## Abstract

**Background:**

Recent evidence shows an association between coronavirus disease 2019 (COVID-19) infection and a severe inflammatory syndrome in children. Cardiovascular magnetic resonance (CMR) data about myocardial injury in children are limited to small cohorts. The aim of this multicenter, international registry is to describe clinical and cardiac characteristics of multisystem inflammatory syndrome in children (MIS-C) associated with COVID-19 using CMR so as to better understand the real extent of myocardial damage in this vulnerable cohort.

**Methods and results:**

Hundred-eleven patients meeting the World Health Organization criteria for MIS-C associated with severe acute respiratory syndrome coronavirus 2 (SARS-CoV-2), having clinical cardiac involvement and having received CMR imaging scan were included from 17 centers. Median age at disease onset was 10.0 years (IQR 7.0–13.8). The majority of children had COVID-19 serology positive (98%) with 27% of children still having both, positive serology and polymerase chain reaction (PCR). CMR was performed at a median of 28 days (19–47) after onset of symptoms. Twenty out of 111 (18%) patients had CMR criteria for acute myocarditis (as defined by the Lake Louise Criteria) with 18/20 showing subepicardial late gadolinium enhancement (LGE). CMR myocarditis was significantly associated with New York Heart Association class IV (p = 0.005, OR 6.56 (95%-CI 1.87–23.00)) and the need for mechanical support (p = 0.039, OR 4.98 (95%-CI 1.18–21.02)). At discharge, 11/111 (10%) patients still had left ventricular systolic dysfunction.

**Conclusion:**

No CMR evidence of myocardial damage was found in most of our MIS-C cohort. Nevertheless, acute myocarditis is a possible manifestation of MIS-C associated with SARS-CoV-2 with CMR evidence of myocardial necrosis in 18% of our cohort. CMR may be an important diagnostic tool to identify a subset of patients at risk for cardiac sequelae and more prone to myocardial damage.

*Clinical trial registration: *The study has been registered on ClinicalTrials.gov, Identifier NCT04455347, registered on 01/07/2020, retrospectively registered.

## Introduction

Recent evidence shows an association between coronavirus disease 2019 (COVID-19) and a severe inflammatory syndrome in children. Clinical presentation is variable and may include general systemic inflammation, toxic shock syndrome and cardiogenic shock [1]. This new syndrome has been named pediatric inflammatory multisystem syndrome, temporally associated with severe acute respiratory syndrome coronavirus 2 (SARS-CoV-2) (PIMS-TS) or multisystem inflammatory syndrome in children (MIS-C) [2–4]. Cardiac involvement has been described in MIS-C patients [5]. The pathophysiology of this subset of SARS-CoV-2-linked illness is not fully understood, as it appears after the acute infection and is often difficult to detect by pharyngeal swabs. Preliminary findings from imaging and immunological testing may suggest a cytokine storm with a vasculitic process with possible microvascular disease [6].

Cardiovascular magnetic resonance (CMR) imaging allows non-invasive assessment of myocardial inflammation as defined by the Lake Louise Criteria [7]. CMR permits tissue characterization of the entire myocardium and accurate evaluation of ventricular volumes and global and regional ventricular function in children with clinical suspected acute myocarditis [8–11]. Revised LLC proposed in 2018 [12] included also tissue mapping analysis but their applicability in children is limited due to the lack of consistent reference normal values of tissue mapping in pediatric population.

In adults, previous data suggest that up to 20% of hospitalized patients have evidence of cardiac injury [13], but myocarditis, defined on autopsy or endomyocardial biopsy (EMB) according to the Dallas criteria [14] occurs in 4.5% of highly selected cases. In children with COVID-19 and MIS-C, CMR data characterizing myocardial and in general cardiac damage are scarce, limited to small cohorts [5, 15–18].

The aim of this multicenter, international registry is to describe clinical and cardiac characteristics of children hospitalized for MIS-C after confirmed COVID-19 using CMR so as to better understand the full extent of myocardial damage in children.

## Patients and methods

### Study design and patients

This is an observational, international multicenter cohort study of all consecutive patients between March 2020 and April 2021 who met the World Health Organization (WHO) criteria for MIS-C associated with SARS-CoV-2 [4], having cardiac involvement and underwent CMR.

The study was approved by the Research Ethics Board of the Assistance Publique des Hôpitaux de Paris (IRB registration: #00011928, ClinicalTrials.gov Identifier NCT04455347, registered on 01/07/2020, retrospectively registered) and conducted according to MR004 conformity.

### Data collection

Local physicians identified eligible patients and reviewed their medical records. Detailed information on demographic, clinical, biological, thoracic and cardiac imaging (echocardiography, CMR and computed tomomgraphy (CT) scan) features were recorded anonymously on a centralized database. SARS-CoV-2 was defined as “confirmed” by either positive nasopharyngeal polymerace chain reaction (PCR) or positive serology. Cardiac involvement was defined as clinical suspicion of acute myocarditis or left ventricular (LV) ejection fraction (LVEF) < 55% or cardiogenic shock. Myocardial inflammation was diagnosed in the presence of at least two of the Lake Louse Criteria [7] detailed below.

### CMR imaging

CMR studies were performed in each participating center according to clinical indication and locally approved imaging protocols. LV dimensions and function were obtained from a short axis stack using a balanced steady-state free precession images (bSSFP), as well as 2 and 4 chamber views. Black-blood prepared T2-weighted (T2-short tau inversion recovery (STIR)/T2 spin echo) images were acquired along the 4-chamber view, 2-chamber view and the short-axis LV planes. These were followed by contrast-enhanced images. Early gadolinium enhancement (EGE) images were obtained with T1-weighted black-blood prepared spin echo and enhanced cine-bSSFP sequences, acquired along the 4-chamber, 2-chamber and short-axis LV stack. Late gadolinium enhancement (LGE) images were acquired with an inversion recovery gradient echo pulse sequence, 8–12 min after injection of contrast media (0.2 mmol/Kg of gadolinium chelate) in the short axis covering the entire LV from the mitral valve plane to the ventricular apex, as well as 2 chamber and 4-chamber views as indicated on case-to-case basis.

Myocardial inflammation was diagnosed in the presence of at least two of the Lake Louise criteria [7]: (1) evidence of regional or global myocardial edema with T2 hyperintensity (T2 ratio > 2, where T2 ratio = Myocardial signal intensity Skeletal muscle signal intensity); (2) evidence of myocardial hyperemia and capillary leak with EGE on cine bSSFP sequence (visual assessment) or on T1 weighted spin echo imaging with absolute signal intensity increase between pre and post gadolinium images of more than 45% (1,9 ratio) or (3) evidence of myocardial necrosis and fibrosis (visual assessment) with non-ischemic regional distribution with LGE.

The evaluation of EGE was also performed on cine-bSSFP sequence (visual assessment) soon after contrast injection. This method is not part of Lake Louise Criteria but several authors reported its efficacy in evaluating hyperemia during acute myocarditis. [9, 19, 20].

Due to the lack of consistent reference normal values of tissue mapping in children, we did not apply the updated Lake Louise Criteria [12].

### Statistical analysis

Categorical variables were presented as frequencies with percentages and compared by Fisher’s exact test; odds ratios and their 95% confidence interval (95% CI) were computed. Normally distributed continuous variables were reported by mean ± standard deviation and compared by t-test; non-normally distributed continuous variables were presented as medians with interquartile ranges (IQR) and compared using the Wilcoxon rank sum test. Multiple samples were compared by ANOVA or Kruskal–Wallis, as appropriate. Laboratory variables were normalized in order to bring them to a common scale. Logistic regression was performed to identify independent risk factors for myocarditis. Missing data were not included in the analysis, imputations were not performed for missing data. A p-value < 0.05 was considered statistically significant (two-sided). SAS (version 9.4, SAS Institute, Cary, North Carolina, USA) was used for statistical analysis.

## Results

A total of 111 children (42 (38%) female) were included. Detailed presentation of demographic, clinical and laboratory characteristics is summarized in Tables 1, 2, 3. Median age at disease onset was 10.0 years (IQR 7.0–13.8). Comorbidities (pre-existent conditions) were reported in 25/106 (24%) children, most commonly respiratory diseases such as bronchiolitis or asthma. None of the patients had associated pre-existing cardiac pathologies. All children had evidence of recent COVID-infection by either positive nasopharyngeal PCR or SARS-CoV-2 antibodies and fulfilled the WHO criteria for MIS-C. Previous COVID infection was more frequent than acute infection, as shown by the high prevalence of SARS-CoV-2 antibodies (98% of the cohort), with 27% of patients having both antibodies and still positive PCR. All children presented with fever, most had gastrointestinal (89/107, 83%) and/or cutaneous manifestations (70/102, 69%).

**Table 1:** Patient characteristics at baseline

	Total n = 111	Myocarditis (Group 1) (n = 20)	No myocarditis (Group 2) (n = 91)	p-value
Female	42 (38)	7 (35)	35 (38)	0.999
Ethnicity				0.203
Caucasian	36/100 (36)	10/19 (53)	26/81 (32)	
Afro-American	45/100 (45)	5/19 (26)	40/81 (49)	
Asian	8/100 (8)	1/19 (5)	7/81 (9)	
Hispanic	11/100 (11)	3/19 (16)	8/81 (10)	
Age at symptom onset, years	10.0 (7.0–13.8)	10.7 (7.5–16.0)	10.0 (7.0–13.0)	0.370
Comorbidity^a^	25/106 (24)	6/19 (32)	19/87 (22)	0.380
COVID serology positive	96/98 (98)	18/18 (100)	78/80 (98)	0.999
COVID PCR and serology positive	30/111 (27)	4/20 (20)	26/91 (29)	0.611
Duration between symptom onset and hospital admission, days	5 (4–6)	4 (4–6)	5 (4–6)	0.200
Follow-up duration (days)	55 (43–100)	53 (39–81)	58 (44–105)	0.641

Values are presented as absolute numbers (%) or medians (IQR). NA = not available. The denominator for each field is reflective of the total number of available data entry points

^a^Associated comorbidities (cumulative): pulmonary (bronchiolitis, asthma) n = 16, autism n = 2, overweight/obesity n = 8, and history of myocarditis, chronic tuberculosis, psoriasis, arterial hypertension, systemic lupus, congenital adrenal hyperplasia by 21-hydroxylase deficiency, alopecia areata n = 1 each

**Table 2:** Clinical and treatment characteristics

	Total n = 111	Myocarditis (Group 1) (n = 20)	No myocarditis (Group 2) (n = 91)	p-value
Fever	111/11 (100)	20/20 (100)	91/91 (100)	NA
Adenopathy	27/91 (30)	5/15 (33)	22/76 (29)	0.762
Respiratory symptoms	35/97 (36)	8/16 (50)	27/81 (33)	0.257
Gastrointestinal symptoms	89/107 (83)	15/18 (83)	74/89 (83)	0.999
Cutaneous symptoms	70/102 (69)	12/16 (75)	58/86 (67)	0.770
Syncope	1/108 (1)	0/17	1/91 (1)	0.999
Palpitation	13/96 (14)	3/15 (20)	10/81 (12)	0.422
Arrhythmias	7/104 (7)	3/17 (18)	4/87 (5)	0.084
Chest pain	12/94 (13)	4/16 (25)	8/78 (10)	0.118
Dyspnea	34/100 (34)	8/16 (50)	26/84 (31)	0.159
NYHA class I	68/93 (73)	8/15 (53)	60/78 (77)	0.011*
class II	2/93 (2)	0/15	2/78 (3)	
class III	8/93 (9)	0/15	8/78 (10)	
class IV	15/93 (16)	7/15 (47)	8/78 (10)	
Hospitalization ICU	87/111 (78)	15/20 (75)	72/91 (79)	0.765
Arrhythmias during hospitalization	7/86 (8)	3/14 (21)	4/72 (6)	0.082
Respiratory failure	45/105 (43)	7/16 (44)	38/89 (43)	0.999
Circulation failure	69/107 (64)	9/18 (50)	60/89 (67)	0.183
Renal failure	34/104 (33)	2/17 (12)	32/87 (37)	0.051
Treatment				
Inotropic support	71/100 (71)	11/16 (69)	60/84 (71)	0.999
Mechanical support	9/103 (9)	4/17 (24)	5/86 (6)	**0.039**
Ventilation support	40/98 (41)	6/14 (43)	34/84 (40)	0.999
Immunosuppressive therapy during hospitalization	66/90 (73)	12/16 (75)	54/74 (73)	0.999
Corticosteroids	91/109 (83)	17/20 (85)	74/89 (83)	0.999
IVIG	98/109 (90)	17/19 (89)	81/90 (90)	0.999
Antiaggregant therapy	84/106 (79)	14/18 (78)	70/88 (80)	0.999
Anticoagulant therapy	66/105 (63)	12/19 (63)	53/86 (63)	0.999
Death	0	0	0	NA

Bold p values were significative

Values are presented as absolute numbers (%). *Def*  definition, *Hosp*  hospitalization, *ICU* intensive care unit,* IVIG* intravenous immunoglobulin, *NYHA* New York Heart Association. The denominator for each field is reflective of the total number of available data entry points. *NYHA 1 vs NYHA 4 p = 0.005

**Table 3:** Laboratory parameters at admission

	Total n = 111	Myocarditis (Group 1) (n = 20)	No myocarditis (Group 2) (n = 91)	p-value
Hemoglobin (N 12–16 g/L)	11.2 (9.9–12.7)	12.1 (10.5–13.6)	11.1 (9.9–12.4)	0.056
Leucocytes (N 4.5–13 10^9^/L)	18.1 (13.9–25.0)	15.9 (10.6–36.1)	18.2 (14.1–24.0)	0.682
Lymphocytes (N 1.3–6 10^9^/L)	1.5 (0.7–4.2)	2.6 (1.7–7.0)	1.1 (0.6–3.5)	**0.017**
Platelets (N 150–450 10^9^/L)	183 (144–250)	195 (153–262)	176 (129–239)	0.258
CRP (N < 5.0 mg/L)	23.8 (13.8–31.1)	26.2 (11.3–33.6)	23.4 (14.0–30.6)	0.619
BNP (N < 100 ng/L)	932 (268–2556)	1516 (504–2759)	795 (268–2471)	0.697
NT pro-BNP (N < 300 ng/L)	4818 (1067–13,540)	3389 (498–34,389)	4927 (1338–12,571)	0.969
TnT (N < 26 ng/L)	110 (30–335)	210 (60–340)	104 (23–287)	0.145
TnT peak (N < 26 ng/L)	205 (62–682)	324 (87–1083)	181 (57–540)	0.213

Bold p values were significative

Values are presented as medians (IQR). *CRP*  C-reactive protein, *BNP*  brain natriuretic peptide, *NT pro-BNP*  NT pro brain natriuretic peptide, *TnT* = troponin T

Median duration from symptom onset to hospital admission was 5 days (4–6). Most children (87/111, 78%) were admitted to the pediatric intensive care unit, 71/100 (71%) required inotropic, 40/98 (41%) ventilatory and 9/103 (9%) mechanical support.

Almost half of the patients (45/107, 42%) had a pathologic electrocardiogram (ECG) at admission. Following abnormalities were found in decreasing order: tachycardia (16/21, 76%), repolarization or T-wave abnormalities (20/29, 69%), ST abnormalities (13/24, 54%), low voltage (6/19, 32%) and other rhythm anomalies (6/11, 55%).

Almost all children (107/111, 96%) had echocardiography performed at admission. Mean LVEF at Simpson’s biplane method was 47.7% ± 13.4 and 72/111 (65%) patients had LVEF < 55%.

Only 28/108 (26%) patients had a thoracic CT scan with a median time of 9 days (7–20) after symptom onset. Of them, 16/28 (57%) had pathological findings, most commonly parenchymal lesions (7/23 ground glass opacifications and 5/23 consolidation). Nine children were found to have pleural effusion.

Imaging characteristics are summarized in Tables 4 and 5.

**Table 4:** Echocardiography data

	Total n = 111	Myocarditis (Group 1) (n = 20)	No myocarditis (Group 2) (n = 91)	p-value
LVEF at admission (%)	47.7 ± 13.4	44.6 ± 14.0	48.4 ± 13.2	0.256
LVEF% < 55% (no. of pts, %)	72/111 (65)	15/20 (75)	57/91 (63)	0.438
Lowest LVEF during hospitalisation (%)	42.9 ± 10.9	44.5 ± 12.9	42.5 ± 10.5	0.576
RV dysfunction (no. of pts, %)	18/84 (21)	5/14 (36)	13/70 (19)	0.167
Diastolic dysfunction	10/63 (16)	2/12 (17)	8/51 (16)	0.999
Pericardial effusion (no. of pts, %)	21/99 (19)	3/18 (17)	18/81 (22)	0.756
Coronary dilation (no. of pts, %)	17/98 (17)	1/13 (8)	16/85 (19)	0.456
LVEF at discharge (%)	61 (56–66)	63 (53–68)	61 (56–66)	0.286
LVEF < 55% at discharge (no. of pts, %)	15/71 (21)	3/10 (30)	12/61 (20)	0.431
Duration between first ECHO and ECHO at last follow-up, days	54 (38–123)	54 (46–127)	53 (36–123)	0.571

Values are presented as absolute numbers (%), medians (IQR) or mean (± sd) as appropriate. The denominator for each field is reflective of the total number of available data entry points. *LVEF*  left ventricular ejection fraction, *RV*  right ventricle

**Table 5:** CMR Characteristics at baseline

	Total n = 111	Myocarditis (Group 1) (n = 20)	No myocarditis (Group 2) (n = 91)	p-value
Delay symptom onset-CMR, days (n = 109)	28 (19–47)	27 (17–34)	28 (19–50)	0.330
T2 BB	18/110 (16)	11/20 (55)	7/90 (8)	** < 0.001**
LGE	22/110 (20)	18/20 (90)	0/90	** < 0.001**
LVEF (%)	58 (55–63)	59 (51–63)	58 (55–63)	0.524
LVEF < 55% (no. of pts)	21/105 (20)	5/19 (26)	16/86 (19)	0.527
LVEDVI (ml/m^2^)	77.4 ± 15.3	76.8 ± 15.3	77.6 ± 15.4	0.853
LVESVI (ml/m^2^)	32 (27–38)	34 (25–43)	32 (27–37)	0.464
RVEF (%)	57.3 ± 6.2	56.4 ± 7.8	57.5 ± 5.8	0.519
RVEDVI (ml/m^2^)	74.0 ± 14.3	77.1 ± 12.4	73.2 ± 14.7	0.325
RVESVI (ml/m^2^)	33.2 ± 9.6	35.6 ± 9.8	32.7 ± 9.6	0.290
Pericardial effusion (no. pts)	18/89 (20)	5/10 (50)	13/79 (16)	**0.026**

Bold p values were significative

Values are presented as absolute numbers (%), medians (IQR) or mean (± sd) as appropriate. The denominator for each field is reflective of the total number of available data entry points. *CMR*  cardiac magnetic resonance, *T2 BB*  T2 black blood sequence, *LGE*  late gadolinium enhancement, *LVEF*  left ventricular ejection fraction, *LVEDVI*  left ventricular end diastolic volume indexed for body surface area (BSA), *LVESVI*  left ventricular end systolic volume indexed for BSA, *RVEF*  right ventricular ejection fraction, *RVEDVI*  right ventricular end diastolic volume indexed for BSA, *RVESVI*  right ventricular end systolic volume indexed for BSA, *pts* patients

### CMR diagnosis of myocarditis

Among the 111 children, 20 (18%) were diagnosed with myocarditis (group 1 Myocarditis) based on the Lake Louise criteria.

CMR was performed a median of 28 days (19–47) after symptoms onset, without any difference between patients with (group 1) and without myocarditis (group 2) (p = 0.330).

**Fig. 1:**
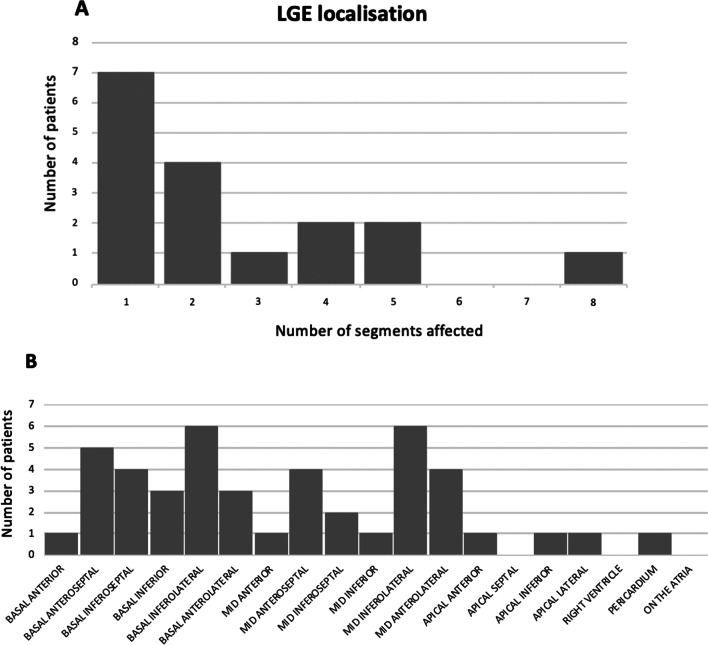
**A** Number of ventricular segments involved per patient with late gadolinium enhancement (LGE) (n = 18) in group 1. **B** Frequency of involvement of different left ventricular segments

**Fig. 2:**
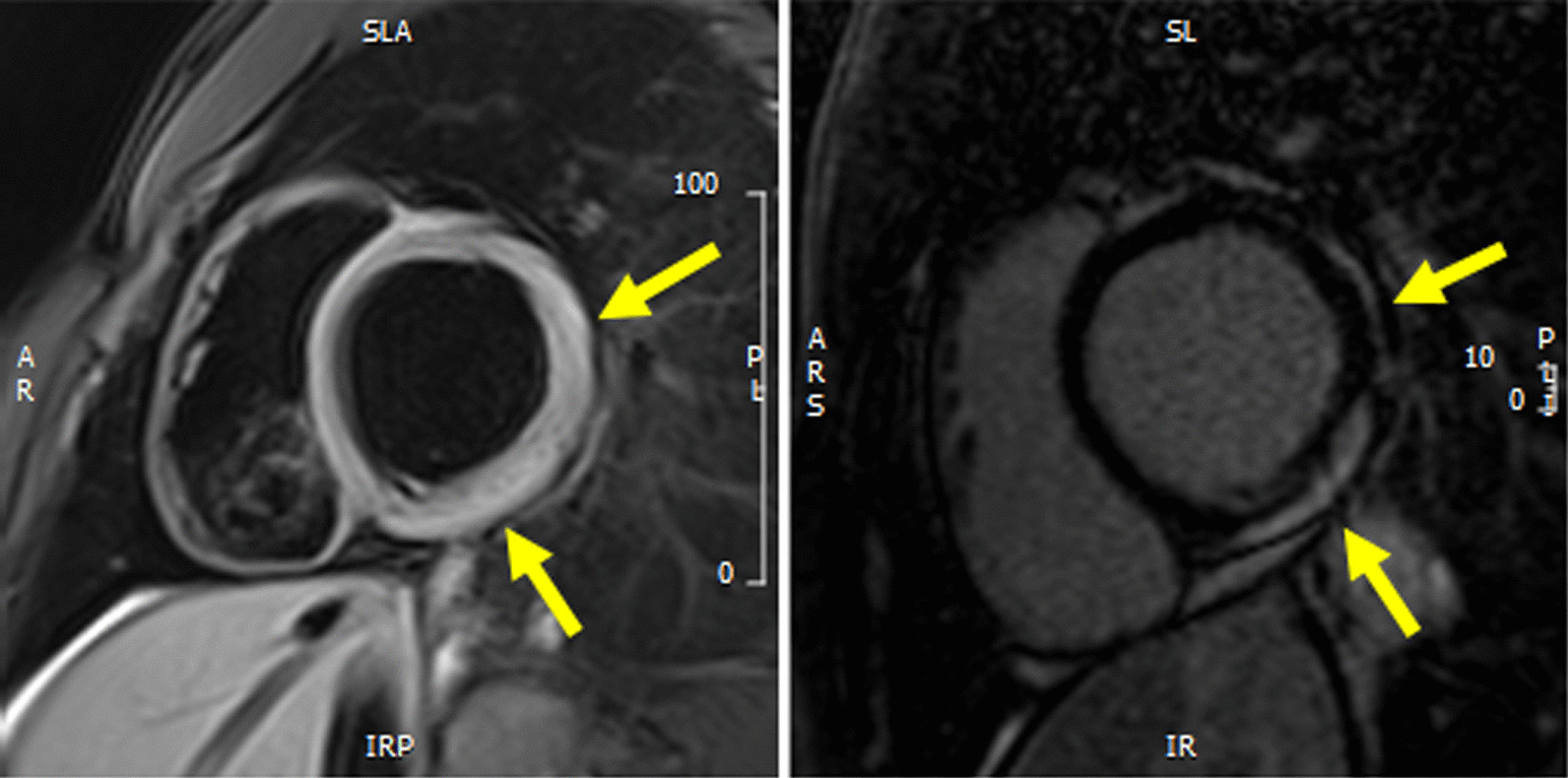
Left panel: short axis view of T2 black blood (BB) acquisition with evidence of oedema in the lateral wall of the left ventricle (yellow arrows). Right panel: short axis view of LGE acquisition with evidence of contrast enhancement in the lateral wall of the left ventricle (yellow arrows)

**Fig. 3:**
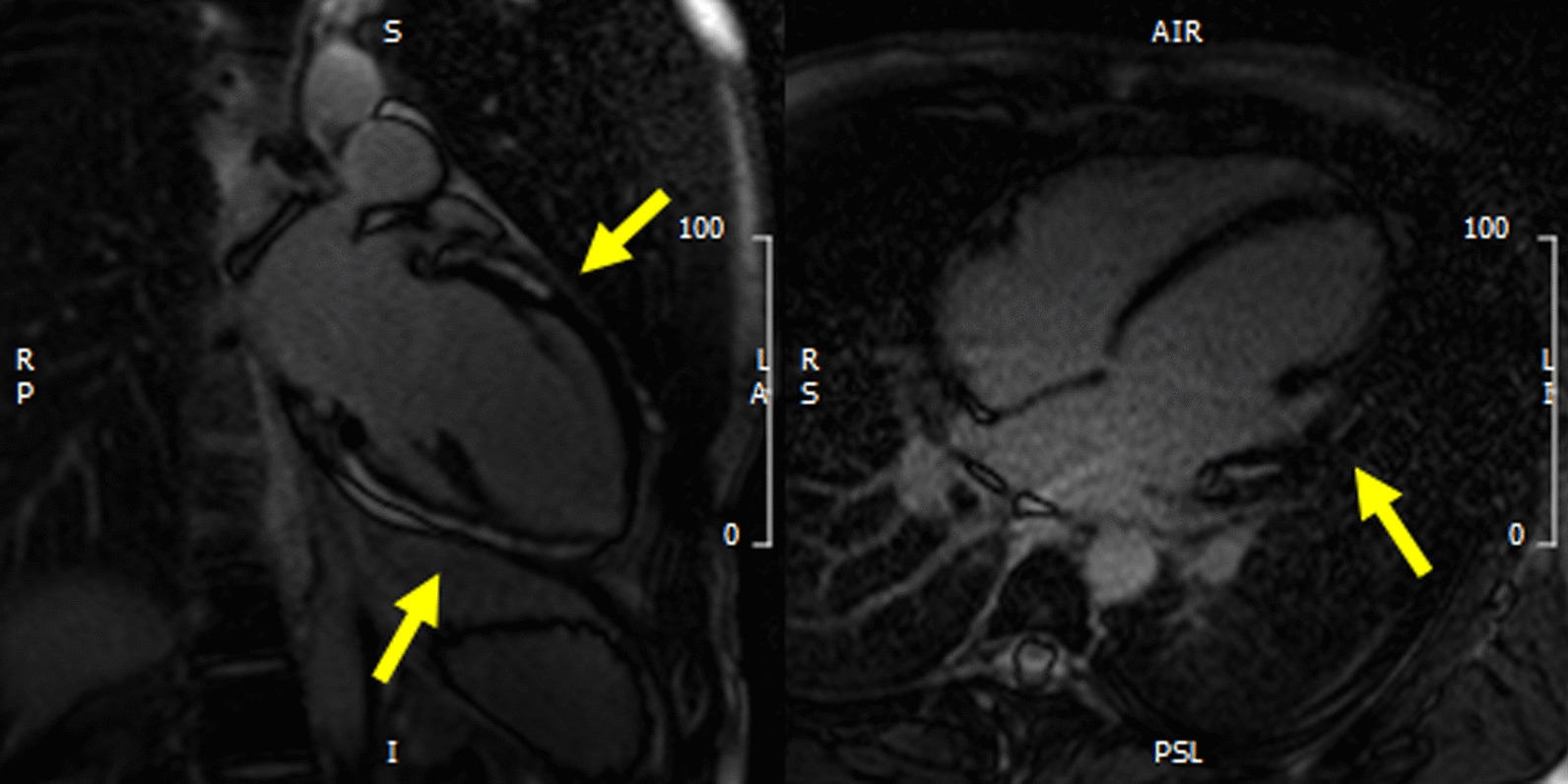
Left panel: two chamber view of LGE acquisition with evidence of contrast enhancement in the inferior and anterior wall of the left ventricle (yellow arrows). Right panel: four chamber view of LGE acquisition with evidence of contrast enhancement in the lateral wall of the left ventricle (yellow arrow)

There was no difference in age at symptom onset. Children in group 1 more commonly presented with New York Heart Association (NYHA) Class 4 (47% versus 10%, p = 0.005, OR 6.56 (95%-CI 1.87–23.00), pairwise comparison with NYHA Class 1). The other pairwise comparisons of the NYHA classes did not find any significant difference between the two groups. During hospital course, children in group 1 more frequently required mechanical support (24% versus 6%, p = 0.039, OR 4.98 (95%-CI 1.18–21.02)). In group 1, 21/24 patients presented with a subepicardial pattern of LGE with 12/20 patients having a multi-segment localization as shown in Figs. 1, 2 and 3.

Regarding laboratory results, lymphocyte counts were significantly higher in group 1 compared to group 2. No other difference in laboratory characteristics was found among the two groups (Table 3).

There was no significant difference in LVEF at diagnosis between the two groups.

In the multivariate regression analysis, NYHA class (p = 0.014), and mechanical support (p = 0.043) remained significantly associated with myocarditis when adjusted for sex and age.

Seventy-nine children (79%) had an echocardiogram performed before discharge, the median discharge LVEF was 61% (56–66) without significant difference between children with and without myocarditis. Among 72 patients with LVEF < 55% at admission (15 in group 1, 57 in group 2), 11 continued to have abnormal function at discharge (1 in group 1, 10 in group 2). Total follow-up duration was 55 (43–100) days. No deaths were reported (Fig. 3).

## Discussion

To the best of our knowledge this is the largest multicenter international cohort of children with strongly suspected or confirmed COVID-19 studied by CMR.

Cardiac involvement has been described in pediatric MIS-C patients in a small group of 20 children [5] and general guidelines about CMR in adult and children during the acute phase of SARS-Cov-2 infection were provided [21]. Moreover, several international alerts were spread during the spring of 2020 about cardiac involvement in children but until now CMR data in large cohorts of children are lacking [22].

We aimed to characterize the myocardial damage caused by COVID-19 using CMR. Our population is primarily made up by MIS-C cases (94%) with evidence of previous SARS-CoV-2 infection and few active viral replications, corroborating the hypothesis that MIS-C is a quite late, immune-mediated complication of viral infection, occurring after the acute phase.

All children were discharged alive with no deaths in our cohort, showing a good prognosis even following severe acute presentation with multi-organ dysfunction.

LV systolic dysfunction by echocardiography was present in the majority of the cohort (65%) with a good short-term prognosis in terms of functional recovery at hospital discharge, without a significant difference between children with and without myocarditis.

Moreover, acute myocarditis, as defined by Lake Louise Criteria, was not frequent, affecting 18% of our cohort, that is at present the largest group of acute phase MIS-C pediatric patients studied by CMR.

In fact, Bartoszek et al. reported normal CMR in 19 children COVID-19 MIS-C with initial LV dysfunction but CMR was realized at 2 months of follow up, not in the acute phase [17]. Webster et al. reported also normal CMR in 20 children without evidence of initial LV systolic dysfunction at 3 months follow up after COVID-19 MIS-C [16], without CMR data during acute phase. Theocharis et al. reported acute phase CMR data in 20 MIS-C patients, with evidence of inflammation in some cases but with no case clearly fulfilling Lake Louise Criteria criteria for acute myocarditis [5].

In our cohort, factors that were significantly associated with myocarditis presentation were NYHA class IV and the need for mechanical support. Due to the small number of patients with myocarditis, more elaborate statistical analysis such as the development of a predictive score could not be performed.

Interestingly, levels of troponin were not associated with myocarditis diagnosis, maybe revealing a non-specific role of troponin in this subset of patients, in agreement with recently reported data by Rajpal et al. [23].

We were not able to identify other clinical, biological, and radiographic differences between the two groups. We therefore speculate—without definitive proof of our data—that the myocarditis phenomenon is part of the same general pathological mechanisms of SARS-CoV-2 infection, with a more specific target of the immunomodulated response localized in the heart as a secondary bystander in susceptible individuals. However, our sample size may lack power to detect small differences between the two groups.

Even if the majority of patients presented with reduced LV systolic function, cardiac involvement in terms of myocardial inflammatory compromise as defined by Lake Louise Criteria was not frequent. We speculate that the majority of patients had a transient inflammation without any lasting cardiac tissue compromise or that LV dysfunction was related to hemodynamic causes associated to MIS-C conditions, such as low diastolic pressure and reduced coronary perfusion.

However, 18/20 (90%) children of group 1 had CMR evidence of myocardial necrosis as demonstrated by the presence of LGE. In this group only one child did not recover LV systolic function at discharge, confirming that LGE is not necessarily associated with persistent LV systolic dysfunction and therefore only CMR examination may evocate its presence [10]. Therefore, CMR was useful to identify patients with myocardial compromise that will therefore require cardiological follow-up.

Integrating our results to existing data [5, 16, 17], we may affirm that acute myocarditis as defined by Lake Louise Criteria in pediatric patients with COVID 19 MIS-C is present in a minority of cases, explaining the negative results of CMR studies in smaller groups. Follow-up CMR data are needed to understand the significance of LGE during acute phase and its prognostic role in follow-up, to estimate the prevalence of permanent versus reversible damage and also to help in defining clinical work-up in children after MIS-C with initial cardiac compromise.

### Limitations

This study is a retrospective observational multicenter cohort study, with all associated limitations. In some instances, not all the variables were available from contributing centers. In these instances, the total number of datasets is reflected in the denominator in the results tables. However, a recommended CMR protocol was distributed, which allowed for more consistent imaging data. The limited parametric mapping data available and also the lack of standardized pediatric normative data made use of the updated Lake Louise Criteria impractical. Because parametric mapping may be more sensitive for the diagnosis of myocarditis, it is possible that some cases of myocarditis were missed using the original Lake Louise Criteria. Moreover, we should consider that hospitalized patients, as in our series, represent the cohort with the most severe presentation of COVID-19 infection, excluding from our observation less severe forms.

## Conclusion

No CMR evidence of myocardial damage was found in most (82%) of our MIS-C cohort, even though about 65% had depressed LVEF at admission. Nevertheless, acute myocarditis, as defined by Lake Louise Criteria, is a possible manifestation of MIS-C associated with COVID-19 with CMR evidence of myocardial necrosis in 18% of our cohort. However, independent of the presence of CMR signs of acute myocarditis, most children demonstrate rapid normalization of cardiac function. CMR may be an important diagnostic tool to identify a subset of patients at risk for cardiac sequelae and more prone to myocardial damage, requiring cardiological follow-up.

## Data Availability

The datasets generated during and/or analysed during the current study are not publicly available due to legislation about medical data privacy but are available from the corresponding author on reasonable request, in the respect of legal obligations.

## Abbreviations

BNP: Brain natriuretic peptide
BSA: Body surface area
bSSFP: Balanced steady-state free precession images
CMR: Cardiovascular magnetic resonance
COVID-19: Coronavirus disease 2019
CRP: C-reactive protein
CT: Computed tomography
ECG: Electrocardiogram
EGE: Early gadolinium enhancement
EMB: Endomyocardial biopsy
ICU: Intensive care unit
LGE: Late gadolinium enhancement
LV: Left ventricle/left ventricular
LVEDVI: Left ventricular end diastolic volume indexed for BSA
LVEF: Left ventricular ejection fraction
LVESVI: Left ventricular end systolic volume indexed for BSA
MIS-C: Multisystem inflammatory syndrome in children
NT pro-BNP: NT PRO Brain natriuretic peptide
NYHA: New York Heart Association
PCR: Polymerace chain reaction
RV: Right ventricle/right ventricular
RVEDVI: Right ventricular end diastolic volume indexed for BSA
RVEF: Right ventricular ejection fraction
RVESVI: Right ventricular end systolic volume indexed for BSA
SARS-CoV-2: Severe acute respiratory syndrome coronavirus 2
STIR: Short tau inversion recovery
TnT: Troponin T
WHO: World Health Organization
